# *Paecilomyces lilacinus *fungaemia in an AIDS patient: the importance of mycological diagnosis

**DOI:** 10.12669/pjms.304.4937

**Published:** 2014

**Authors:** Chuan Hun Ding, Mohd Nizam Tzar, Md Mostafizur Rahman, Najihan Abdul Samat Muttaqillah, Shazatul Reza Mohd Redzuan, Petrick Periyasamy

**Affiliations:** 1 Chuan Hun Ding, Department of Medical Microbiology & Immunology, Universiti Kebangsaan Malaysia Medical Centre, Jalan Yaacob Latif, Bandar Tun Razak, 56000 Kuala Lumpur, Malaysia.; 2 Mohd Nizam Tzar, Department of Medical Microbiology & Immunology, v Universiti Kebangsaan Malaysia Medical Centre, Jalan Yaacob Latif, Bandar Tun Razak, 56000 Kuala Lumpur, Malaysia.; 3 Md Mostafizur Rahman, Department of Medical Microbiology & Immunology, Universiti Kebangsaan Malaysia Medical Centre, Jalan Yaacob Latif, Bandar Tun Razak, 56000 Kuala Lumpur, Malaysia.; 4 Najihan Abdul Samat Muttaqillah, Department of Medical Microbiology & Immunology, Universiti Kebangsaan Malaysia Medical Centre, Jalan Yaacob Latif, Bandar Tun Razak, 56000 Kuala Lumpur, Malaysia.; 5 Shazatul Reza Mohd Redzuan, Department of Medicine, Universiti Kebangsaan Malaysia Medical Centre, Jalan Yaacob Latif, Bandar Tun Razak, 56000 Kuala Lumpur, Malaysia.; 6 Petrick Periyasamy, Department of Medicine, Universiti Kebangsaan Malaysia Medical Centre, Jalan Yaacob Latif, Bandar Tun Razak, 56000 Kuala Lumpur, Malaysia.

**Keywords:** AIDS, *Paecilomyces lilacinus*, Fungaemia, Amphotericin B, Voriconazole

## Abstract

Fungaemia due to *Paecilomyces lilacinus* is generally not considered in AIDS patients because this condition is not categorised as an AIDS-indicator illness. We report a case of a 25-year-old lady who presented to our hospital with Guillain-Barré Syndrome, with the subsequent development of refractory fungaemia, multi-organ failure and disseminated intravascular coagulopathy. Amphotericin B was given as empirical antifungal therapy. HIV screening was reactive and *Paecilomyces lilacinus* was isolated from her blood. The fungaemia did not resolve after one week of amphotericin B treatment. The addition of itraconazole was also unsuccessful in clearing the fungaemia. Accurate mycological diagnosis is important in the care of AIDS patients with fungaemia because of the risk of treatment failure with empirical therapy.

## INTRODUCTION

*Paecilomyces lilacinus* is a saprophytic fungus with a worldwide distribution that is commonly found in soil.^1^ Potential resistance to sterilisation and the ability to colonise clinical materials (catheters and plastic implants) increase the clinical significance of this fungus in both immunocompromised and immunocompetent hosts.^2^ From our observation, patients with human immunodeficiency virus (HIV) infection are prone to various opportunistic fungal infections, namely penicilliosis, histoplasmosis and cryptococcosis. Thus in Universiti Kebangsaan Malaysia Medical Centre (UKMMC), amphotericin B is given as empirical and definitive therapy for invasive fungal infections. *Paecilomyces lilacinus *is an emerging fungal pathogen known for its low in-vitro susceptibility to many conventional antifungal drugs.^3^

## CASE REPORT

This is a case of a 25-year-old housewife who was referred to UKMMC for further investigation and management of her neurological symptoms. The patient became ill four years ago with frequent nose bleeds and joint pains although no medical treatment was sought. Just prior to her presentation to UKMMC, she had pancytopaenia and elevated liver enzymes following a workup for non-specific complaints. She was presumed to have idiopathic thrombocytopaenic purpura (ITP) and was commenced on prednisolone. However, over three weeks her condition worsened with the development of neurological symptoms. Specifically, she developed bilateral lower limb numbness, gait difficulties, upper limb stiffness, droopy eyelids, slurred speech and dysphagia.

On examination, the patient was alert with a Glasgow Coma Scale of 15/15. She was afebrile with bilateral ptosis and ophtalmoplegia. There was mild hepatomegaly and oral candidiasis. She was areflexic and had down going plantar reflexes but sensation in all limbs was intact. 

Laboratory examination showed bicytopaenia (haemoglobin of 9.5 g/dL and platelet count of 50 x 10^9^ cells/L). The anaemia was normocytic and normochromic. The total white cell count was normal (8.8 x 10^9^ cells/L). Her serum alanine transaminase level was raised (171 U/L), and there was hypoalbuminaemia (20 g/L). Serum creatine kinase was highly elevated (2259 U/L). Blood electrolytes were normal, as was the coagulation profile. A computed tomography scan of the brain showed generalized cerebral atrophy. Retroviral disease screening was reactive. The CD4 cell count was 1/mm^3^ and the CD8 count was 66/mm^3^. A nerve conduction study revealed generalized axonal sensory motor polyneuropathy. Lumbar puncture was not performed due to thrombocytopaenia.

She was diagnosed as HIV associated Guillain-Barré Syndrome (GBS) of the acute motor-sensory axonal neuropathy (AMSAN) variant. For the HIV infection, she was started on oral Tenofovir 300 mg daily, oral Emtricitabine 200 mg daily and oral Efavirenz 600 mg daily. For GBS, intravenous immunoglobulin and oral prednisolone 40 mg BD were administered.

Five days after presenting to UKMMC, she was transferred to intensive care due to hypotension and respiratory distress. She also developed metabolic acidosis and fever. The patient was covered empirically for sepsis with intravenous vancomycin 500 mg BD and intravenous imipenem 1g QID. Peripheral blood sample sent for bacteriological culture was found negative for bacteria but there was growth of fungal elements and a diagnosis of sepsis secondary to fungaemia was made.

**Fig.1:**
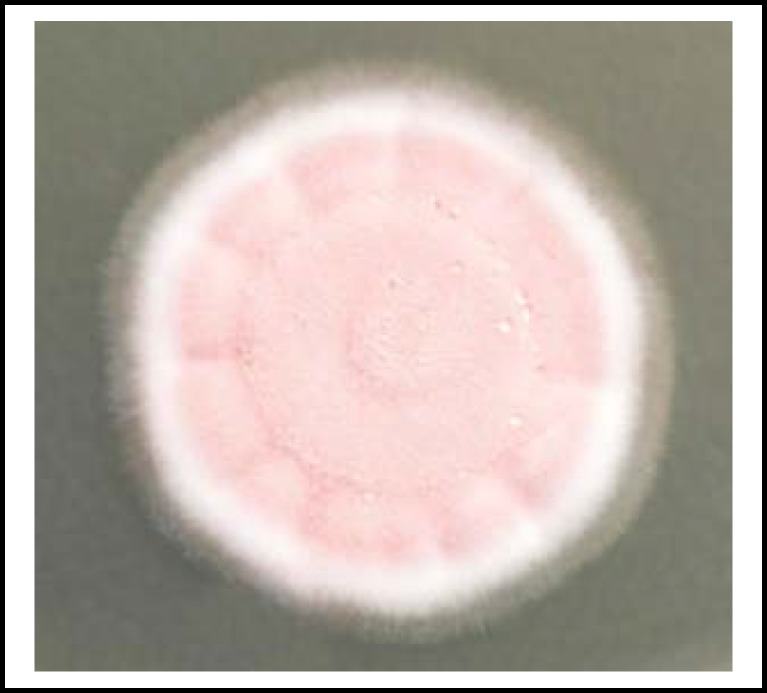
Five-day-old velvety pink colony of *Paecilomyces lilacinus *on Sabouraud dextrose agar

**Fig.2:**
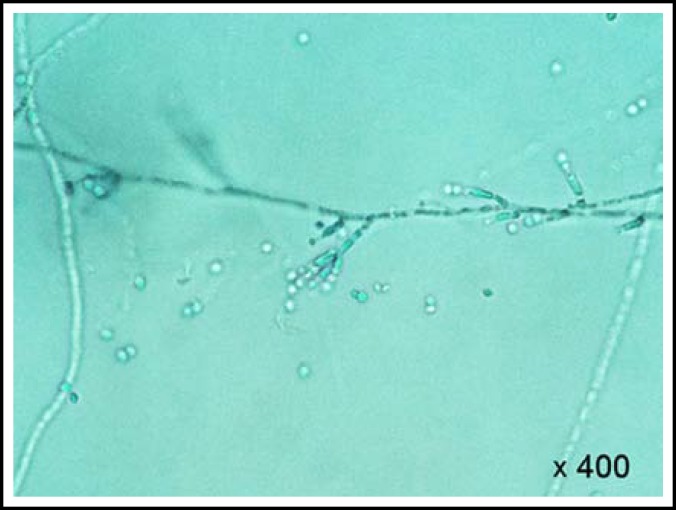
Lactophenol cotton blue stain of *Paecilomyces lilacinus *from slide culture

Intravenous amphotericin B 0.7mg/kg was started while awaiting definitive culture identification of the fungus. By the time the fungus was identified as *Paecilomyces lilacinus *(Fig. 1 and 2), she was already on amphotericin B for a week.

Because *Paecilomyces lilacinus *is known to be resistant in-vitro to amphotericin B, a recommendation to commence voriconazole treatment was made. However, because the co-administration of efavirenz and voriconazole is contraindicated, syrup itraconazole 200 mg TDS was added to the treatment regimen instead. After two weeks in intensive care, the patient developed multiorgan dysfunction syndrome and disseminated intravascular coagulopathy and succumbed to her illness. The final blood culture taken just before she passed away still grew *Paecilomyces lilacinus*.

## DISCUSSION

*Paecilomyces lilacinus* infections mostly manifest as either oculomycoses or cutaneous infections,^2^ neither of which were apparent in our patient. In South East Asia, *Penicillium marneffei* infection is an AIDS-indicator illness with cases being reported from Malaysia since 1995.^4^ In northern Thailand, disseminated penicilliosis marneffei is the third most common opportunistic infection in HIV patients.^5^ Thus, when our patient’s blood specimen had fungal elements, a decision was made to treat her empirically with amphotericin B which is the preferred treatment for acute *Penicillium marneffei* infection.

Since fungaemia is related to the use of central venous catheters,^2^ it is likely that our patient acquired the infection during her stay in hospital, when invasive medical devices were used for central venous pressure monitoring and for the administration of medications, blood products etc.

*Paecilomyces lilacinus* has high minimum inhibitory concentrations (MICs) towards conventional antifungal agents (namely amphotericin B, fluconazole and flucytosine) and most studies also report a high MIC towards itraconazole.^2^ However, despite the high MIC, cases of successful treatment of *Paecilomyces lilacinus* fungaemia with amphotericin B (with or without itraconazole) have been reported in the literature.^2^ Voriconazole, a relatively newer azole, was evaluated as a treatment option and the MIC was found to be low.^1^ Thus, while there are no optimal treatment recommendations for *Paecilomyces lilacinus* infections, voriconazole appears to be the most effective agent.^2^

The co-administration of standard doses of voriconazole (i.e. at 200 mg BD) and efavirenz (i.e. at 600 mg daily) was previously contraindicated because this resulted in a significant decrease in voriconazole levels and an increase in efavirenz levels.^6^ Our patient was given efavirenz as part of her anti-retroviral treatment regime even before fungal culture results were available. When healthy volunteers were given efavirenz and voriconazole at various dose combinations, it was found that by doubling the voriconazole dose and halving the efavirenz dose, the pharmacokinetic interaction resulting from co-administration of these drugs could be compensated.^6^ Although extrapolating data from studies on healthy subjects to HIV-infected patients may have limitations,^7^ the dosage adjustments proposed may be attempted if the fungaemia does not resolve on amphotericin B.

Once *Paecilomyces lilacinus* is identified in a clinical specimen, the ideal duration of antifungal administration is unknown. It is likely that a protracted course of therapy is necessary and for individuals on anti-retroviral treatment, antifungal therapy should be given until partial recovery of CD4 counts. While the CD4 threshold above which therapy may be discontinued for *Paecilomyces lilacinus* infections has not been reported, for penicilliosis marneffei the CD4 threshold is > 100/mm^3^.^8^ Thus it may be reasonable to adopt a CD4 threshold of > 100/mm^3^ for invasive *Paecilomyces lilacinus* infections as well although formal studies on this are needed.

In conclusion, when fungaemia is present in AIDS patients with indwelling medical devices, it is important to consider *Paecilomyces* *lilacinus *as one of the potential pathogens. Amphotericin B is often chosen as empirical therapy for fungaemia due to its broad spectrum but fungal cultures are mandatory for accurate species identification and to guide definitive therapy using voriconazole.
